# P-1623. Implementation of MRSA Nasal PCR Pharmacy Protocol on Vancomycin Use for SSTI

**DOI:** 10.1093/ofid/ofae631.1790

**Published:** 2025-01-29

**Authors:** Sarah Withers, Abigale Lipe, Caroline Jozefczyk, Carmen M Faulkner-Fennell, Danielle Eskens

**Affiliations:** Prisma Health, Greenville, South Carolina; Prisma Health Upstate, Greenville, South Carolina; Prisma Health - Upstate, Greenville, South Carolina; Prisma Health-Upstate, Greer, South Carolina; Prisma Health Upstate, Greenville, South Carolina

## Abstract

**Background:**

Recent published literature has shown that methicillin-resistant *Staphylococcus* aureus (MRSA) polymerase chain reaction (PCR) nasal swabs have similar utility for skin and soft tissue infections (SSTI) to well-established pneumonia data with a specificity ranging from 92.2-94% and negative predictive value ranging from 92-94%. The purpose of this study was to evaluate the effect of implementing a pilot pharmacy-led protocol for ordering MRSA PCR nasal swabs in vancomycin-treated SSTI.

**Methods:**

A single-center, pre- and post-implementation observational study was conducted on adult floor patients admitted to a single institution between March 2023 to January 2024 who were on vancomycin for only the indication of SSTI. Exclusion criteria included Gram positive organism in blood culture, admission to any ICU, or if the suspected infection was a deep surgical site infection, necrotizing fasciitis, osteomyelitis, or a vascular graft infection. The primary outcome of duration of vancomycin therapy was compared between pre- and post-implementation groups. Secondary outcomes included length of hospitalization, acceptance rate of de-escalation, total duration of antibiotic therapy, SSTI-related readmissions within 30 days, and MRSA PCR statistics.

**Results:**

A total of 229 patients were included in the study with 160 patients in the pre-protocol group and 70 patients in the post-protocol group. 52.4% of patients were male and 78.2% of patients were white with majority of SSTI being lower extremity cellulitis. There was an increase in MRSA PCR collection from 14.4% to 48.6% in the post-protocol group and in MRSA PCR pharmacy interventions from 0.6% to 28.6%. Duration of vancomycin therapy was similar between both groups and the average total duration of antibiotic therapy was longer than current guideline recommendations for SSTI.

**Conclusion:**

While there was an increase in MRSA PCR nasal swab usage after piloting a new MRSA PCR protocol, the PCR results did not have a significant change on antibiotic utilization for skin and soft tissue infections. As a result of this study, alternative stewardship efforts have been identified for skin and soft tissue infection management.

**Disclosures:**

**All Authors**: No reported disclosures

